# Emotion Regulation and Self-Harm Among Forensic Psychiatric Patients

**DOI:** 10.3389/fpsyg.2021.710751

**Published:** 2021-08-24

**Authors:** Natalie Laporte, Stéphanie Klein Tuente, Andrejs Ozolins, Åsa Westrin, Sofie Westling, Märta Wallinius

**Affiliations:** ^1^Child and Adolescent Psychiatry, Department of Clinical Sciences Lund, Lund University, Lund, Sweden; ^2^Centre for Ethics, Law and Mental Health, Institute of Neuroscience and Physiology, The Sahlgrenska Academy, University of Gothenburg, Gothenburg, Sweden; ^3^Department of Research, Regional Forensic Psychiatric Clinic, Växjö, Sweden; ^4^Department of Psychology, Linnaeus University, Växjö, Sweden; ^5^Department of Clinical Sciences Lund, Psychiatry, Lund University, Lund, Sweden; ^6^Office for Psychiatry and Habilitation, Psychiatric Clinic Lund, Region Skåne, Lund, Sweden; ^7^Office for Psychiatry and Habilitation, Psychiatry Research Skåne, Region Skåne, Lund, Sweden

**Keywords:** emotion regulation, self-harm, non-suicidal self-injury, forensic psychiatry, difficulty in emotion regulation scale, inventory of statements about self-injury scale

## Abstract

Emotion regulation has been specifically linked to both non-suicidal self-injury (NSSI) and attempted suicide. It is also known that self-harm is disproportionally higher (30–68.4%) in forensic samples than in the general population, yet knowledge about the association between emotion regulation and self-harm in forensic settings is scarce. The purpose of this study was to describe emotion regulation in a sample of forensic psychiatric patients, to explore dimensions and levels of emotion regulation between forensic psychiatric patients with and without self-harm, and to explore associations between forensic psychiatric patients’ self-reported emotion regulation and self-reported functions of NSSI. A cohort of forensic psychiatric inpatients (*N*=98) was consecutively recruited during 2016–2020 from a high-security forensic psychiatric clinic in Sweden. Data were collected through the self-report measures Difficulties in Emotion Regulation Scale (DERS) and Inventory of Statements About Self-injury (ISAS). In relation to the first aim, median total and subscales scores for DERS were reported. Results showed a statistically significant difference in emotion regulation between participants with and without self-harm (*p*=0.004), with a medium effect size (Cohen’s *d*=0.65) for the DERS total scale. The DERS subscales returned large differences for Impulse (*p*=0.001, *d*=0.86), Goals (*p*=0.014, *d*=0.58), and Strategies (*p*=0.012, *d*=0.54) between participants with and without self-harm. Finally, DERS scores were correlated with both the interpersonal (*r_s_*=0.531, *p*<0.001, *n*=43) and intrapersonal factors (*r_s_*=0.503, *p*<0.001, *n*=43) of NSSI as reported on the ISAS. Participants with self-harm (NSSI and/or suicide attempts) demonstrated significantly more difficulties with emotion regulation than those without self-harm. Emotion dysregulation was associated with both interpersonal and intrapersonal functions of NSSI in the participants. We suggest further studies on forensic psychiatric patients’ maladaptive behaviors that focus on substance abuse, self-harm, and aggressive behaviors in relation to the regulation and expression of emotion.

## Introduction

Emotion regulation is the mechanism through which individuals modify their emotions to achieve a certain goal (Aldao et al., 2010) or to enhance and/or inhibit their emotional experiences and expressions (Gross, 2002; Bridges et al., 2004; Rottenberg and Gross, 2007; Calkins and Mackler, 2011). Emotion regulation develops over a lifetime, beginning with emotion recognition (Yoo et al., 2006). Young children who have not yet developed emotion recognition skills depend on their caregivers to teach them how to recognize and interpret emotions (Rothbart et al., 1992; Cassidy, 1994). As children develop, their emotional skills evolve from recognition to internalization and self-regulation (Zeman and Shipman, 1996; Denham, 1998) and continue to develop during adolescence (Zeman et al., 2006). Previous studies have demonstrated that emotion regulation is vital for mental health, while maladaptive emotion regulation (or emotion dysregulation) is associated with the development and maintenance of a wide range of mental disorders (Aldao et al., 2010; Gratz and Tull, 2010), such as substance use (Kun and Demetrovics, 2010), anxiety disorders (Cisler et al., 2010), and borderline personality disorder (Gratz et al., 2006). Maladaptive and destructive behaviors, including aggression toward oneself and others, have also been associated with emotion dysregulation (Buckholdt et al., 2009; Mikolajczak et al., 2009; Roberton et al., 2012, 2014).

Emotion regulation can generally be described as a continuum ranging from under-regulation, through intermediate adaptive regulation, to over-regulation (Roberton et al., 2012, 2014). Both under-regulation and over-regulation of emotions can be defined as maladaptive, and their expression negatively affects the individual’s functioning and wellbeing. Under-regulation is more easily recognized because it leads to overt behaviors corresponding to the person’s immediate feelings. An individual with under-regulated emotions might, for example, express their present anger by slamming a door, shouting, or exhibit other kinds of aggression. Over-regulated emotions, however, are harder to pinpoint, because the person tries to avoid showing the feelings and is more likely to withdraw from a situation than engage in it. Although over-regulating emotions might seem beneficial in the moment, it can increase the likelihood of later maladaptive behavioral expressions such as aggressive outbursts as the emotions are deflected rather than processed in the moment they arise (Roberton et al., 2012, 2014), and the individual’s suffering is not decreased by such delay.

Emotion dysregulation has been specifically studied and linked to self-harm (Buckholdt et al., 2009; Mikolajczak et al., 2009). Self-harm is a broad term that includes both non-suicidal self-injury (NSSI) and self-inflicted harm with the intention of committing suicide (suicide attempt; Hawton, 2002). The *Diagnostic and Statistical Manual of Mental Disorders, 5th edition*, defines NSSI as “the deliberate, self-inflicted destruction of body tissue without suicidal intent and for purposes not socially sanctioned, include[ing] behaviors such as cutting, burning, biting and scratching skin” [American Psychiatric Association (APA), 2013]. NSSI behaviors range from those mentioned to swallowing objects and self-strangulation to forms of severe and permanent physical injury, including eye-gouging, genital mutilation, and amputations (e.g., ears or tongue; see items included in ISAS). Studies on the functions of self-harm, focused mainly on NSSI, have proposed two functional domains: interpersonal and intrapersonal. Interpersonal functions are actions aimed to gain social-positive reinforcement rewards, such as increased attention from friends, family, or caregivers (Nock, 2008). Intrapersonal functions, in contrast, are a means to avoid, reduce, or eliminate unwanted emotional responses (Chapman et al., 2006) or their overwhelming, uncontrollable, and intensely painful effects (Linehan, 1993). Previous clinical, empirical, and theoretical work has indicated that NSSI is primarily used as an emotion regulation strategy (e.g., Linehan, 1993; Gratz, 2003; Chapman et al., 2006; Kleindienst et al., 2008). Studies have found that NSSI may be used mainly to relieve negative affect states with high arousal, such as frustration, feeling overwhelmed, or high anxiety, or low arousal states as sadness, emptiness, or loneliness (Klonsky, 2009). Reductions in negative affect have been found to predict lifetime frequency of cutting, indicating that affective changes associated with NSSI could further reinforce the behavior (Klonsky, 2009). However, existing research on the functions of NSSI is based mostly on clinical observations and self-reports from self-harming clients; researchers have previously described the difficulties of categorizing the functions of NSSI (Gratz, 2003). In a recent study of forensic psychiatric patients, intrapersonal functions, such as self-punishment, affect regulation, and marking distress, were the most relevant to the participants (Laporte et al., 2021).

An extensive systematic review has shown that self-harm and/or suicide attempts frequently co-occur with aggressive behaviors toward others (O’Donnell et al., 2015). This is of specific interest in forensic psychiatric settings since many forensic psychiatric patients have a history of aggressive behaviors. Individuals displaying aggressive behaviors toward others may therefore also be considered at risk for self-directed aggression (O’Donnell et al., 2015). It is known that suicide attempts are disproportionally higher among forensic samples than among the general population (Liebling, 1993). A 2002 study by Brooker et al. (2002) and data from the current sample (detailed in Laporte et al., 2021) show prevalence rates of self-harm and/or suicide attempts ranging from 30% in prison settings to 68.4% in forensic mental health settings. However, although various NSSIs may seem to be similar in their methods and intentions, a deeper investigation may reveal varying underlying mechanisms and motivations that are not immediately obvious. To provide adequate assessment and treatment, it is crucial to understand the mechanisms and motivations underlying NSSI.

The evidence to date indicates both an association between emotion regulation and NSSI in different samples and a higher prevalence of the behavior in forensic psychiatric patients. Knowledge on the relation between emotion regulation and NSSI in a forensic psychiatric sample could provide important information for assessing risks, formulating treatment goals, and monitoring treatment progress. Researchers in forensic psychiatry have only recently increased their interest in emotion regulation, however, and studies on emotion regulation in forensic populations remain scarce (Roberton et al., 2012; García-Sancho et al., 2014; Garofalo et al., 2016; Garofalo and Velotti, 2017). Increased knowledge on NSSI and its functions in relation to emotion regulation seems especially important in forensic psychiatric settings, to avoid misconceptions of patients’ intent behind NSSI such that the behavior rather functions to manipulate or control the environment instead of being an expression of psychological dysfunction and suffering.

### Aims

The purpose of this study was to describe emotion regulation and its associations with NSSI and suicide attempts among forensic psychiatric patients, with the following specific aims:

1. Describe emotion regulation among forensic psychiatric patients;
2. Compare dimensions and levels of emotion regulation between forensic psychiatric patients with and without NSSI and/or suicidal behavior; and
3. Explore associations between forensic psychiatric patients’ self-reported emotion regulation and self-reported function of NSSI.

## Materials and Methods

### Participants

All patients who met the initial criterion of being cared for at a high-security forensic psychiatric clinic in Sweden during the data collection period of November 2016 to November 2020 were candidates for participation. To be included, patients had to have a predicted stay of more than 8 weeks at the clinic and be able to fulfill the tasks in the study without an interpreter. All patients were assessed by their treating psychiatrist prior to participation and were excluded if assessed as unable to provide informed consent. At the current clinic, the median length of care was 46 months for patients with ongoing care during 2020, in comparison with 59 months for forensic psychiatric patients in Sweden in general during the same period (Swedish National Forensic Psychiatric Register, RättspsyK, 2020). The general treatment plan at the clinic included psychopharmacological treatment (predominantly antipsychotics), psychological treatment, occupational therapy, physiotherapy, social interventions, and general risk management.

The aim was to collect 100 participants, but due to the COVID-19 pandemic, inclusion of participants was terminated in November 2020 after 98 patients had participated (56% participation rate). The participants mean age was 34.9years (range 19–62, *SD*=10.7), and the majority were male (86.7%; *n*=85) and had been born in Sweden (71.4%; *n*=70; Laporte et al., 2021). The most common, current mental disorders were within the spectrum of schizophrenia or other psychotic disorders, with substance-related and addictive disorders as common comorbidity. The criminological background of the participants consisted of different violent offenses, with repeated occasions of drug offenses and theft or robbery along with other crimes. For detailed information on inclusion, length of stay, previous forensic psychiatric care, and psychosocial and clinical characteristics of this sample, see Laporte et al. (2021). During data collection, nine participants chose to terminate their participation before all data had been collected, and one self-report was assessed as unreliable. The characteristics of the nine patients who chose to terminate their participation were 90% male, and all had different current primary diagnoses and index crimes. Because participants had been told they could terminate their participation at any time without giving a cause, no reason for dropout were available.

### Procedures

All eligible participants received information on the study from the first author or a fellow Ph.D. student, both of whom had clinical experience in working with forensic psychiatric patients. After receiving oral and written information on the study, patients who agreed to participate provided their written, informed consent. Following this, participants answered self-rating questionnaires and semi-structured questions about suicide attempts. A data collector was present when participants answered the questionnaires to provide emotional or practical support if needed. After each participant completed the questionnaires, the data collector and a senior clinician and researcher in the field reviewed and assessed the quality of the data. Participants received a small monetary compensation for taking part in the study.

### Measures

#### Self-Harm

Information on lifetime NSSI was collected from files and self-reports and complemented by interviews. Information on suicide attempts (any attempt, age at onset, violent attempts, and risk of completed suicide at most serious attempt) was collected separately. In total, more than half of the participants (*n*=67; 68.4%) had at some point engaged in some kind of self-harm, with banging one’s head or fist against a wall or other solid surface and cutting as most common method of NSSI, and hanging as most common method for attempted suicide (Laporte et al., 2021). Thus, two groups were established: forensic psychiatric patients with self-harm (*n*=67) and forensic psychiatric patients without self-harm (*n*=31).

The self-report instrument Inventory of Statements About Self-injury (ISAS; Klonsky and Glenn, 2009) was used to assess the functions of NSSI. The ISAS assesses NSSI in two parts: the frequency of 12 NSSI behaviors done intentionally but without suicidal intent and 13 theoretical functions of NSSI. This study used only the second part of ISAS. Information on the first part is detailed in Laporte et al. (2021). Participants who confirmed one or more NSSI behaviors in Part 1 were asked to proceed to Part 2. The second part scores the 13 potential functions of NSSI (affect regulation, anti-dissociation, anti-suicide, autonomy, interpersonal boundaries, interpersonal influence, marking distress, peer bonding, self-care, self-punishment, revenge, sensation seeking, and toughness) by three items per function rated as “0 = not relevant,” “1 = somewhat relevant,” or “2 = very relevant.” Scores for each function can range from 0 to 6. In Klonsky and Glenn (2009), the ISAS factors had excellent internal consistency and expected correlations with both clinical and contextual factors, and these findings support the reliability and validity of ISAS. The Swedish version of ISAS demonstrates good internal consistency for both interpersonal and intrapersonal factors (Lindholm et al., 2011). Internal consistency for the ISAS self-report items in this study was good (*α*=0.898 for the intrapersonal scale and *α*=0.859 for the interpersonal scale). After dropouts, analyses of the ISAS self-reports were based on 43 participants.

In this study, a suicide attempt is defined as a “nonfatal self-directed potentially injurious behavior with any intent to die as a result of the behavior. A suicide attempt may or may not result in injury” (Crosby et al., 2011, p. 21). In the Results section, we chose to specify suicide attempts, because we believe this is of clinical relevance. Participants were asked, “Have you ever made a suicide attempt with the intention to die?” Participants who answered “Yes” were asked to report their most recent method of suicide attempt, if any attempt of suicide had been made during the last 6months, and substance-related events connected to the attempt, and the potential lethality of the latest attempt.

In this manuscript, the term self-harm refers to NSSI and/or suicide attempts. The term NSSI refers specifically to non-suicidal self-injuries and excludes intentional suicide attempts.

#### Emotion Regulation

Emotion regulation was assessed through the Difficulties in Emotion Regulation Scale (DERS), a 36-item self-report that was developed to comprehensively assess emotion dysregulation in six domains: (1) non-acceptance of negative emotions (Non-acceptance) (2) inability to engage in goal-directed behaviors when distressed (Goals) (3) difficulties controlling impulsive behaviors (Impulse) (4) limited access to emotion regulation strategies perceived as effective (Strategies) (5) lack of emotional awareness (Awareness), and (6) lack of emotional clarity (Clarity). Participants were asked to indicate how often the items applied to themselves by rating their answer on a 5-point Likert-type scale (1 = almost never, 2 = sometimes, 3 = half of the time, 4 = mostly, and 5 = almost always). Total DERS scores range from 36 to 180. The DERS has been found to demonstrate good test–retest reliability and adequate construct and predictive validity (Gratz and Roemer, 2004; Gratz and Tull, 2010). Internal consistency in the current sample was good for the total scale (*α*=0.93) and subscales (*α*=0.60–0.89). Gillespie et al. (2018) used the DERS self-report in an offender population and reported similar internal consistency (*α*=0.66–0.86) for the six scales. After dropouts, analyses on DERS self-reports were based on 88 participants.

#### Statistical Methods

For the first aim, descriptive and frequency tables were used to report descriptive statistics for DERS total and subscale scores. For the second aim, Welch’s *t*-tests were used due to skewed data distributions. Effect sizes were tested using Welch’s *d*. A binary variable called self-harm was created by merging two variables (suicide attempt yes/no and NSSI behavior yes/no). For the third aim, bivariate correlations using Spearman’s rho (*r_s_*) were performed to examine associations between functions of NSSI as measured by the ISAS factors and emotion regulation as measured by the DERS subscales. All statistical analyses were performed using Jamovi software and IBM SPSS 25. Analyses for the third aim included only those participants who answered both DERS and ISAS self-reports. For the three participants who failed to answer one to three items in the DERS self-report, a mean score for the specific subscale was individually imputed.

#### Ethical Considerations

The studied population is considered to be especially vulnerable; ethical considerations are therefore highly relevant. The treating forensic psychiatrist was consulted before any candidate for participation was informed about the study. Candidates considered as not currently suitable for the study due to psychiatric status (e.g., acute psychosis or imminent risk of violence) or inability to provide informed consent were excluded. All participants were informed about their right to terminate participation at any time without any reason required. If the participants had questions concerning their participation, they could at any time contact the responsible researchers. Emotional support was available 24/7 from ward staff. The study, including the monetary reward (which was low in order not to give an incentive that would compromise the free consent), was approved by the Research Ethics Committee at Linköping University, Dnr 2016/213–31 and 2017/252–32.

## Results

### Emotion Regulation in Forensic Psychiatric Patients

**Figure 1:**
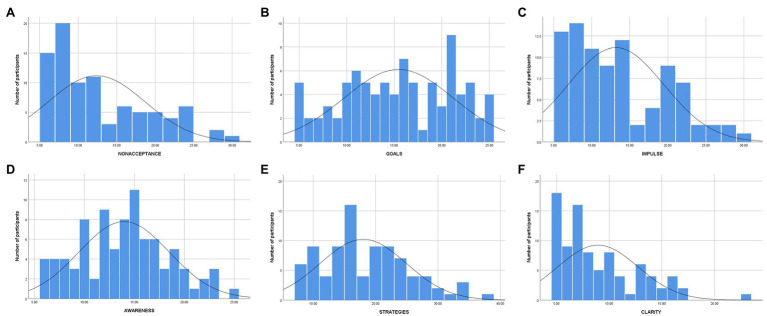
Distribution of DERS subscale scores in forensic psychiatric patients (*n* =88). ^*^ **(A)** = Non-acceptance; **(B)** = Goals; **(C)** = Impulse; **(D)** = Awareness; **(E)** = Strategies; and **(F)** = Clarity.

**Figure 2:**
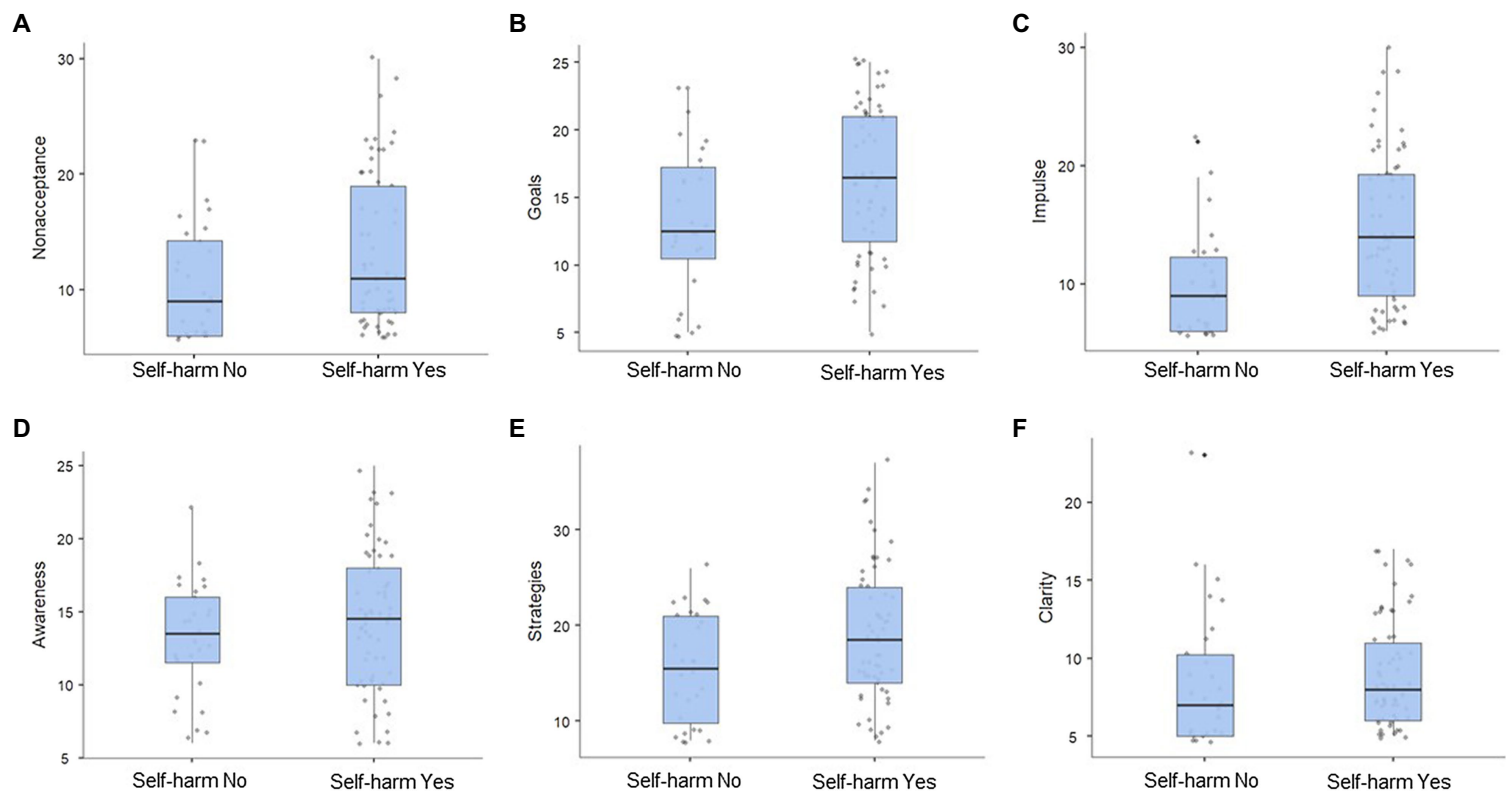
Distribution of median values on the DERS subscales in forensic psychiatric patients with (*n* =67) and without self-harm (*n* =61). ^*^ **(A)** = Non-acceptance; **(B)** = Goals; **(C)** = Impulse; **(D)** = Awareness; **(E)** = Strategies; and **(F)** = Clarity.

Table 1 presents descriptive DERS scores for the entire cohort, with both mean and median values to facilitate comparisons to other samples. As presented in Figure 1, providing histograms for all the DERS subscales in the whole cohort, the distribution of the majority of the subscale scores in this sample was highly skewed. Although the general trend skewed negative, with many participants reporting scores in the lower region except for the Goals and (less so) the Awareness subscales, the responses ranged widely. For distribution of DERS scores in the participants with and without self-harm, see Figure 2.

**Table 1:** Descriptive statistics on DERS scores in forensic psychiatric patients (*n*=88).

DERS scales	M	*Mdn*	*SD*	*IQR*	Range
DERS Total	81.9	76.5	25.5	41.5	36–140
Non-acceptance	12.4	10	6.3	10	6–30
Goals	15.4	16	5.8	10	5–25
Impulse	13.2	12	6.3	11.75	6–30
Awareness	14.0	14	4.5	7	6–25
Strategies	18.0	16.5	6.9	9.75	8–37
Clarity	8.9	8	3.8	5	5–23

### Dimensions and Levels of Emotion Regulation in Relation to Self-Harm in Forensic Psychiatric Patients

Comparing results in participants with and without self-harm showed a statistically significant difference in emotion regulation between the two groups (*p*=0.004), with a medium effect size (Cohen’s *d*=0.65) for the DERS total scale. Participants who reported self-harm had a median DERS total score of 85 (*IQR*=47.5), while the group without reported self-harm had a median value of 71.1 (*IQR*=29.25). The DERS subscales showed a large difference in the subscale Impulse between participants with (*Mdn*=14, *IQR*=10.75) and without self-harm (*Mdn*=9, *IQR*=6.75, *p*<0.001) with a large effect size (Cohen’s *d*=0.86). See Table 2 for details. In general, scores for both groups ranged widely over the DERS subscales (see Figure 2).

**Table 2:** Levels of emotion dysregulation in relation to self-harm in forensic psychiatric patients (*n* =88)^*^.

Subscales of DERS	95% Confidence interval				
	*p*	Mean difference	Lower	Upper	Cohen’s *d*
Non-acceptance	0.068	−2.417	−5.02	0.183	−0.388
Goals	0.014	−3.250	−5.81	−0.690	−0.583
Impulse	<0.001	−5.033	−7.37	−2.701	−0.855
Awareness	0.211	−1.207	−3.11	0.700	−0.268
Strategies	0.012	−3.648	−6.46	−0.835	−0.544
Clarity	0.698	−0.371	−2.29	1.549	−0.097

^*^ *Non-parametric t-test performed due to non-normal distribution of scores in the sample.*

### Dimensions of Emotion Regulation and Function of NSSI in Forensic Psychiatric Patients

The DERS total scale was positively correlated with both the ISAS interpersonal (*r_s_*=0.531, *p*<0.001, *n*=43) and intrapersonal factor (*r_s_*=0.503, *p*<0.001, *n*=43) with large effect sizes. Table 3 illustrates the bivariate correlations between the DERS subscales and the two ISAS factors, demonstrating higher scores on several DERS subscales as related to the function of NSSI as measured by the ISAS factors, all with medium to large effect sizes, for participants with NSSI. In particular, the Awareness and Clarity subscales were neither statistically significant nor meaningfully related to the functions of NSSI.

**Table 3:** Associations (Spearman’s rho) between DERS subscales and ISAS factors in forensic psychiatric patients (*n* =43).

		DERS subscales						
		Non-acceptance	Goals	Impulse	Awareness	Strategies	Clarity
Intrapersonal ISAS	*r_s_*	0.519	0.363	0.516	−0.042	0.374	0.268
	*p*	0.000	0.017	0.000	0.790	0.013	0.082
Interpersonal ISAS	*r_s_*	0.398	0.406	0.542	0.150	0.389	0.243
	*p*	0.008	0.007	0.000	0.336	0.010	0.117

This analysis included only participants who had participated in both the DERS and ISAS self-reports.

## Discussion

This study reports levels and dimensions of emotion (dys-) regulation in a sample of forensic psychiatric patients in relation to NSSI, suicide attempts, and the functions of NSSI. Group comparisons indicated elevated levels of emotion dysregulation on several subscales of the DERS among participants with existing or former self-harm (NSSI and/or history of suicide attempt) compared with participants without a history of self-harm. Scores on emotion regulation were associated with both the interpersonal and intrapersonal functions of NSSI in forensic psychiatric patients.

### Emotion Regulation in Forensic Psychiatric Patients

In the studied sample, the DERS scores were comparable to those reported in earlier studies on community samples (Neumann et al., 2010; Garofalo et al., 2018) and male violent offenders (Garofalo et al., 2018). Previously, DERS scores have been found to be elevated among individuals with psychiatric disorders and symptoms considered to be characterized by emotion dysregulation (e.g., bipolar disorder, post-traumatic stress disorder, and panic attacks) as well as generalized anxiety disorder (Salters-Pedneault et al., 2006). In a sample such as this, with complex psychiatric comorbidities and histories of aggressive and antisocial behaviors (Laporte et al., 2021), difficulties with emotion regulation could be expected to be demonstrated by higher DERS scores. However, this was not the case in the current study, and the DERS scores were remarkably similar to those previously reported for male violent offenders (Garofalo et al., 2018). There are several possible explanations for this.

First, individuals’ perceptions of their own emotion regulation skills might be similar across samples, while mentalization and expressions of emotion regulation differ. For example, individuals residing in forensic settings may demonstrate difficulties in being able to reflect and think about their own feelings and emotions, which eventually may lead to emotions becoming overwhelming and expressed in disruptive behaviors (Velotti and Garofalo, 2015). Second, a major concern that arose during the data collection in the current study was whether DERS captures the full construct of emotion regulation in forensic samples, given the complex interactions of psychiatric comorbidities and cognitive deficits (Garieballa et al., 2006; Puzzo et al., 2019). The DERS has been used in prison samples (e.g., Garofalo and Velotti, 2017; Garofalo et al., 2018), but forensic psychiatric patients demonstrate even more complex needs than prison population, and this could affect the applicability of the DERS or other self-reporting instruments of emotion regulation. Observations from this particular data collection suggest some participants (especially those with intellectual challenges or on the autism spectrum) had difficulty understanding the nuances of some items and needed assistance from the data collector. In addition, the referential perspectives of forensic psychiatric inpatients (e.g., what does it really mean to be aware of one’s feelings?) may differ from those in other samples. The lack of a normative sample for the DERS further hampers interpretation and generalization of the results.

In any case, emotion regulation or dysregulation seems crucial in forensic psychiatric settings where patients demonstrate overt and self-directed aggression in the form of deliberate self-harm, previously linked to deficient emotion regulation (Gratz and Roemer, 2008; Roberton et al., 2015; Scott et al., 2015; Garofalo et al., 2016; Velotti et al., 2016). In a sample of violent offenders, Garofalo and Velotti (2017) found a significant relationship between emotion dysregulation and negative emotionality with aggressive tendencies. They also showed that adequate emotion regulation skills – especially the ability to control one’s behavior under negative emotional arousal – might buffer a positive relationship between negative emotionality and aggression. Roberton et al. (2014) described three emotion regulation skills as relevant in relation to disruptive behaviors in the form of aggression: emotional awareness, acceptance of emotions, and access to different emotion regulation strategies. Thus, by practicing awareness and acceptance of emotions, and learning a variety of emotion regulation strategies, individuals in forensic psychiatry could achieve increased skills in emotion regulation that might be helpful in the management of disruptive behaviors of different kinds, not only aggression.

### Dimensions and Levels of Emotion Regulation in Relation to Self-Harm in Forensic Psychiatric Patients

In line with previous research on emotion regulation (Chapman et al., 2006; Gratz and Roemer, 2008; Gratz et al., 2012), levels of emotion dysregulation were statistically significantly higher in participants with self-harm. Specifically, participants with self-harm reported more difficulties than those without in engaging in goal-directed behaviors, controlling impulsive behaviors, and accessing effective emotion regulation strategies, although the confidence intervals were quite large. This is consistent with previous findings in non-clinical samples that individuals with co-occurring, clinically relevant, maladaptive behaviors, such as self-harm and eating disorders, scored higher for difficulties with emotion regulation on both the DERS total scores and several subscales than individuals without those behaviors (Buckholdt et al., 2015). The same study, using a clinical sample, also reported that individuals with co-occurring self-harm, eating disorders, and substance misuse had more difficulties with emotion regulation (DERS total score and subscales) than those with only substance misuse. Taken together, this could indicate that individuals with multiple maladaptive behaviors or disorders have more difficulty regulating their emotions than those with a single maladaptive behavior. This was not studied specifically in the current study but would seem relevant for forensic psychiatric patients demonstrating complex comorbid psychopathologies and a variety of maladaptive behaviors.

In the specific DERS subscales related to self-harm and/or suicide attempts in this sample of forensic psychiatric patients, impulsivity has long been discussed as an underlying factor in non-fatal suicide attempts and self-harm (Mann et al., 2009; McHugh et al., 2019). Impulsivity is the tendency to react rapidly with no plan when exposed to internal or external stimuli, with little or no consideration of the negative effects of these reactions on oneself or others (Moeller et al., 2001). Individuals who engage in self-harm have been found to be slower to respond appropriately to stimuli in response inhibition tasks than controls (McHugh et al., 2019). A possible deficit in response inhibition, as evidenced in increased impulsivity, could explain why these participants engage in self-harm: It requires more mental effort for them to stop themselves from reacting self-destructively than to give in to the impulse. Recently, it was demonstrated that a subsample of this current group of forensic psychiatric patients (male participants with a violent history) showed more disinhibitory behaviors than controls. Their higher level of disinhibition was associated with slower response and slower neural information processing during a response inhibition task, suggesting less efficient neural information processing in these patients (Delfin et al., 2020). Although the current results cannot corroborate this suggestion, they do indicate that the ability to refrain from impulsive behaviors is especially reduced in forensic psychiatric patients who self-harm. However, the overall levels of emotional dysregulation in our sample were not different from those reported for samples from the general population. This raises a question about which factors, such as context or general difficulties regulating inhibition, might moderate the effect of emotion dysregulation on disruptive behaviors (e.g., self-harm). Furthermore, it is important to keep in mind that the DERS Impulse Scale cannot be translated to impulsivity commonly measured in impulse control tasks, but rather reflects emotional impulsivity presented as difficulties in refraining from impulsive reactions when emotionally upset, sometimes referred to as negative urgency (see, e.g., Smith and Cyders, 2016; Garofalo and Wright, 2017; Feil et al., 2020). Yet, our findings imply that individuals with dysfunctional inhibition skills might have greater difficulty regulating their behavior in general, not only their self-harming. This remains an interesting area for future studies, where in-depth analyses on mechanisms (e.g., in relation to clinical characteristics in patients) need to be performed.

As mentioned, participants with self-harm and/or suicide attempts showed higher DERS scores on subscales measuring their inability to engage in goal-directed behaviors when distressed and limited access to effective emotion regulation strategies. Taken together, this indicates a specific need in forensic psychiatric patients who self-harm to learn and practice effective strategies of emotion and behavior regulation. Skills training in these areas is a vital component of several established treatment methods directed to individuals with emotion regulation difficulties and subsequent disruptive behaviors, such as dialectical behavior therapy (McCann et al., 2000; Brown et al., 2013) or emotion regulation group therapy (Gratz and Gunderson, 2006; Gratz and Tull, 2011). Recently, the concept of eHealth has gained increasing support in forensic psychiatry (Kip et al., 2018), where developments in virtual reality technology for skills training (e.g., aggression management; Tuente et al., 2018; Klein Tuente et al., 2020) have been especially promising. However, while these could provide completely new arenas for skills training in emotion and behavior regulation for forensic psychiatric patients, there remains a great need for research on effective treatment components.

Interestingly, participants with and without self-harm and/or suicide attempt did not differ on the subscales Awareness and Clarity. This suggests that those participants had no less emotional awareness or clarity than those without self-harm and/or suicide attempt and raises the question of whether people who self-harm perceive themselves as aware and clear about their emotions at the time, but are nevertheless unable to refrain from maladaptive behaviors. Another explanation, as previously mentioned in another part of this discussion, is that the items on these scales place too high a demand on forensic psychiatric patients’ abstract thinking, especially since cognitive deficits have been found to be common in such groups (e.g., Puzzo et al., 2019). This in turn raises problems in relation to the use of self-reports in forensic psychiatric settings, which are further complicated by the general low reading level in such contexts (Svensson et al., 2015).

### Dimensions of Emotion Regulation and Function of Self-Harm in Forensic Psychiatric Patients

In this study, emotion dysregulation was strongly associated with both the interpersonal and intrapersonal functions of NSSI. Research on interpersonal functions of self-harm is generally focused on adolescents and young adults rather than forensic samples (e.g., Gratz, 2003). However, several interpersonal functions of self-harm in forensic populations, relevant to the current findings, have previously been suggested. The forensic environment is often characterized by loss of liberty, limited access to activities, lack of personal space, and other restrictions, all of which are social factors that could incite individual disruptive behaviors (Lanza et al., 1994). In addition to the possible difficulties with emotion regulation in patients, the forensic context presents a challenging situation. Patients in these settings might inflict NSSI to create or reinforce interpersonal connections with others (e.g., caregivers), especially if their more adaptive modes of communication are reduced (Nock, 2008; Turner et al., 2012). In this study, we found moderate to strong associations between the interpersonal functions of NSSI and the dimensions of emotion regulation, specifically regarding (in)ability to engage in goal-directed behaviors when distressed, control of impulsive behaviors, and access to effective emotion regulation strategies. That is, in the assessment and management of NSSI in forensic populations, strategies for emotion regulation and impulse control need to be considered in relation to the communicative aspect of NSSI.

Furthermore, the intrapersonal functions of NSSI were, to a moderate to strong degree, associated with emotion dysregulation as measured on several of the DERS subscales in this sample. Self-harm, along with other maladaptive behaviors, such as disordered eating and substance abuse, has been proposed as an emotion regulation and coping strategy to avoid feelings, such as hopelessness, anger, or stress (Sherwood et al., 2000; Chapman et al., 2006; Klonsky, 2007; Jutengren et al., 2011). In forensic psychiatry, where patients may be reluctant to express strong emotions due to feared consequences (e.g., coercive measures and increased medication), NSSI might function both as a maladaptive coping strategy to handle intense emotions through deflection and as a means of self-punishment (Podvoll, 1969; Klonsky, 2007; Daffern and Howells, 2009). Emotional relief has been reported as the single most common reason for NSSI (Brown et al., 2002). Although the current results cannot be generalized to assert that NSSI functions as emotional relief in forensic psychiatric patients, it is obvious that these people are in a difficult situation with both environmental limitations and emotional challenges that require them to learn and use functional coping strategies. Given the current results, it seems that NSSI had both interpersonal and intrapersonal functions in this sample, governed and affected by the individuals’ emotion regulation. However, the motives and functions of behavior, such as NSSI, might not necessarily be consistent. For instance, although a behavior might be motivated only by the wish to release negative emotions (intrapersonal function), it might also have the unexpected function of attracting caregivers’ attention (interpersonal function). This interaction should be investigated further, preferably through single-case studies with in-depth interviews, to provide a more comprehensive understanding.

Lastly, there were no clinically or statistically significant relations between the functions of NSSI and the DERS subscales Awareness and Clarity. As mentioned previously, individuals in forensic psychiatric settings might find the items in these scales difficult to interpret and answer. Since this issue has not been investigated in other studies, future research should endeavor to replicate the findings and determine the psychometric properties of the DERS in forensic psychiatric samples.

### Clinical Implications and Future Research

To the best of our knowledge, the current study is the first to investigate emotion regulation in relation to NSSI and its functions in forensic psychiatric patients. Therefore, our findings, albeit with the limitations described below, have potentially important clinical implications and could be used in the design of future studies on the subject. First, forensic psychiatric patients with self-harm differ in emotion regulation from their counterparts without these behaviors. Treatment interventions might thus be improved by targeting patients’ emotional and behavioral regulation skills. Although this might be especially relevant for patients with self-harm, such skills are fundamental for any individual’s everyday functioning, and a possible target group might therefore be a broader group of forensic psychiatric patients with disruptive behaviors possibly linked to deficits in emotion regulation and coping. Based on our findings, an important aspect to consider would be managing impulsivity, both emotional and behavioral, in forensic psychiatric patients; more studies in this area are needed.

Second, the DERS may be suboptimal for assessing emotion regulation in forensic psychiatric patients. Future research should investigate methods of assessing emotion regulation in forensic psychiatric settings considering the heterogeneous groups’ complex psychopathologies and cognitive deficits and possibly making use of recent developments in virtual reality.

Third, the results indicate that the function of NSSI varies among forensic psychiatric patients. Attention to, and intervention in, NSSI must be provided to all forensic psychiatric patients, regardless of whether they present more other-directed or self-directed aggression. NSSI is a prominent risk factor for suicide attempts (Klonsky et al., 2013; Muehlenkamp et al., 2018). It has been demonstrated that suicide-related mortality is higher in forensic samples than in the general population (Fazel et al., 2005, 2011; Carson and Cowhig, 2020). In prison populations, self-harm has been found to precede suicide in 43 to 50% of cases (Dooley, 1990; Fazel et al., 2008). We suggest that future research on emotion dysregulation among forensic psychiatric patients examines multiple maladaptive behaviors or disorders (e.g., aggressive behaviors, eating disorders, and substance use) and not only self-harm, especially since individuals with co-occurring maladaptive behaviors have a higher degree of emotional dysregulation than those with only one of these maladaptive behaviors (Gratz et al., 2012). In general, there are several possible reasons, motivations, or functions underlying NSSI, creating a challenge both for staff members’ understanding of the behavior and for the design of interventions. Since individual acts of NSSI may have different explanations at different times for an individual, these multiple and sometimes conflicting reasons can also make it difficult for individuals to communicate the intention of their NSSI when seeking help. For this reason, we believe it is important to always listen to the individuals’ explanations and expressions of feelings after each incident of NSSI even though the reasons may differ. We suggest single-case studies for a better understanding of the causes of these behaviors among forensic patients.

### Strengths and Limitations

This study has several strengths, such as data from a relatively large forensic psychiatric patient sample residing in a high-security setting, and the contribution to the field of forensic psychiatry of unique knowledge on NSSI, attempted suicide, and emotion regulation. Nevertheless, the results should be seen in the light of several limitations. First, data were collected at one time point in a retrospective, cross-sectional design, so firm conclusions cannot be drawn regarding causality between emotion regulation and NSSI. Second, the study included self-report questionnaires, known to be inherently susceptible to biases, such as social desirability, in which participants report what they perceive to be suitable under the circumstances (Vigil-Colet et al., 2012). It should also be pointed out that the participants in the current study suffered from severe mental disorders (Laporte et al., 2021) that could have affected their ability to interpret and understand specific items in the DERS. Third, because the sample consisted of forensic psychiatric patients treated at a high-security forensic psychiatric hospital in Sweden, some of whom had been referred from other forensic psychiatric caregivers due to difficulties with their care, specific needs, or behaviors (e.g., aggression), they might not represent forensic psychiatric patients in general and the generalizability of the results is therefore limited. Fourth, participants were a heterogeneous group presenting with a wide variety of self-harming behaviors ranging from minor non-suicidal self-injuries all the way through to serious suicide attempts. In the future research, we suggest that studies of self-harm in forensic samples consider the severity and frequency of deliberate self-harm in relation to emotion regulation.

## Conclusion

In this study, we conclude that dysfunctional emotion regulation in a sample of forensic psychiatric patients can be associated with both general self-harming behavior and the interpersonal and intrapersonal functions of NSSI. This information is highly relevant to clinical settings and should improve the understanding of self-harm as a more complex phenomenon with more nuanced functions than the current clinical view of such actions as communicative, attention-seeking, or efforts to relieve anxiety.

## Data Availability Statement

The raw data supporting the conclusions of this article will be made available by the authors, without undue reservation.

## Ethics Statement

The studies involving human participants were reviewed and approved by Research Ethics Committee at Linköping University, 2016/213-31 and 2017/252-32. The patients/participants provided their written informed consent to participate in this study.

## Author Contributions

NL and MW developed the study concept. AO, SW, and ÅW contributed to the study design. Data collection and data analysis were performed by NL. NL and SK drafted the paper. MW, AO, SW, and ÅW provided critical revisions. All authors approved the final version of the paper for submission.

## Conflict of Interest

The authors declare that the research was conducted in the absence of any commercial or financial relationships that could be construed as a potential conflict of interest.

## Publisher’s Note

All claims expressed in this article are solely those of the authors and do not necessarily represent those of their affiliated organizations, or those of the publisher, the editors and the reviewers. Any product that may be evaluated in this article, or claim that may be made by its manufacturer, is not guaranteed or endorsed by the publisher.

## References

[ref1] Aldao A. Nolen-Hoeksema S. Schweizer S. (2010). Emotion-regulation strategies across psychopathology: a meta-analytic review. Clin. Psychol. Rev. 30, 217–237. 10.1016/j.cpr.2009.11.004, PMID: 20015584

[ref2] American Psychiatric Association (APA). (2013). Diagnostic and Statistical Manual of Mental Disorders, 5th *Edn*. Washington, DC: APA Press.

[ref3] Bridges L. J. Denham S. A. Ganiban J. M. (2004). Definitional issues in emotion regulation research. Child Dev. 75, 340–345. 10.1111/j.1467-8624.2004.00675.x, PMID: 15056188

[ref4] Brooker C. Repper J. Beverley C. Ferriter M. Brewer N. (2002). Mental Health Services and Prisoners: A Review. Sheffield: School of Health and Related Research, University of Sheffield.

[ref5] Brown J. F. Brown M. Z. Dibiasio P. (2013). Treating individuals with intellectual disabilities and challenging behaviors with adapted dialectical behavior therapy. J. Ment. Health Res. Intellect. Disabil. 6, 280–303. 10.1080/19315864.2012.700684, PMID: 23914278 PMC3725667

[ref6] Brown M. Z. Comtois K. A. Linehan M. M. (2002). Reasons for suicide attempts and nonsuicidal self-injury in women with borderline personality disorder. J. Abnorm. Psychol. 111, 198–202. 10.1037/0021-843X.111.1.198, PMID: 11866174

[ref7] Buckholdt K. E. Parra G. R. Anestis M. D. Lavender J. M. Jobe-Shields L. E. Tull M. T.. (2015). Emotion regulation difficulties and maladaptive behaviors: examination of deliberate self-harm, disordered eating, and substance misuse in two samples. Cogn. Ther. Res. 39, 140–152. 10.1007/s10608-014-9655-3

[ref8] Buckholdt K. E. Parra G. R. Jobe-Shields L. (2009). Emotion regulation as a mediator of the relation between emotion socialization and deliberate self-harm. Am. J. Orthop. 79, 482–490. 10.1037/a0016735, PMID: 20099939

[ref9] Calkins S. D. Mackler J. S. (2011). “Temperament, emotion regulation, and social development,” in Social Development: Relationships in Infancy, Childhood, and Adolescence. eds. Underwood M. K. Rosen L. H. (New York: Guilford Press), 44–70.

[ref10] Carson E. A. Cowhig M. P. (2020). Mortality in State and Federal Prisons, 2001–2016 Statistical Tables. Washington, DC: US Department of Justice, Office of Justice Programs, Bureau of Justice Statistics.

[ref11] Cassidy J. (1994). Emotion regulation: influences of attachment relationships. Monogr. Soc. Res. Child Dev. 59, 228–249. 10.1111/j.1540-5834.1994.tb01287.x 7984163

[ref12] Chapman A. L. Gratz K. L. Brown M. Z. (2006). Solving the puzzle of deliberate self-harm: the experiential avoidance model. Behav. Res. Ther. 44, 371–394. 10.1016/j.brat.2005.03.005, PMID: 16446150

[ref13] Cisler J. M. Olatunji B. O. Feldner M. T. Forsyth J. P. (2010). Emotion regulation and the anxiety disorders: an integrative review. J. Psychopathol. Behav. Assess. 32, 68–82. 10.1007/s10862-009-9161-1, PMID: 20622981 PMC2901125

[ref14] Crosby A. E. Ortega L. Melanson C. (2011). Self-Directed Violence Surveillance: Uniform Definitions and Recommended Data Elements. *Vol*. 21. Atlanta, GA: Centers for Disease Control and Prevention, National Center for Injury Prevention and Control.

[ref15] Daffern M. Howells K. (2009). The function of aggression in personality disordered patients. J. Interpers. Violence 24, 586–600. 10.1177/0886260508317178, PMID: 18445830

[ref16] Delfin C. Ruzich E. Wallinius M. Björnsdotter M. Andiné P. (2020). Trait Disinhibition and NoGo event-related potentials in violent mentally disordered offenders and healthy controls. Front. Psychol. 11:577491. 10.3389/fpsyt.2020.577491, PMID: 33362599 PMC7759527

[ref17] Denham S. A. (1998). Emotional Development in Young Children. New York: Guilford Press.

[ref18] Dooley E. (1990). Prison suicide in England and Wales, 1972–87. Br. J. Psychiatry 156, 40–45. 10.1192/bjp.156.1.40, PMID: 2256964

[ref19] Fazel S. Benning R. Danesh J. (2005). Suicides in male prisoners in England and Wales, 1978–2003. Lancet 366, 1301–1302. 10.1016/S0140-6736(05)67325-4, PMID: 16214601

[ref20] Fazel S. Cartwright J. Norman-Nott A. Hawton K. (2008). Suicide in prisoners: a systematic review of risk factors. J. Clin. Psychiatry 69, 1721–1731. 10.4088/JCP.v69n1107, PMID: 19026254

[ref21] Fazel S. Grann M. Kling B. Hawton K. (2011). Prison suicide in 12 countries: an ecological study of 861 suicides during 2003–2007. Soc. Psychiatry Psychiatr. Epidemiol. 46, 191–195. 10.1007/s00127-010-0184-4, PMID: 20140663

[ref22] Feil M. Halvorson M. Lengua L. King K. M. (2020). A state model of negative urgency: do momentary reports of emotional impulsivity reflect global self-report? J. Res. Pers. 86:103942. 10.1016/j.jrp.2020.103942, PMID: 32322127 PMC7176315

[ref23] García-Sancho E. Salguero J. M. Fernández-Berrocal P. (2014). Relationship between emotional intelligence and aggression: a systematic review. Aggress. Violent Behav. 19, 584–591. 10.1016/j.avb.2014.07.007

[ref24] Garieballa S. S. Schauer M. Neuner F. Saleptsi E. Kluttig T. Elbert T.. (2006). Traumatic events, PTSD, and psychiatric comorbidity in forensic patients–assessed by questionnaires and diagnostic interview. Clin. Pract. Epidemiol. Ment. Health 2:7. 10.1186/1745-0179-2-7, PMID: 16595005 PMC1488838

[ref25] Garofalo C. Holden C. J. Zeigler-Hill V. Velotti P. (2016). Understanding the connection between self-esteem and aggression: the mediating role of emotion dysregulation. Aggress. Behav. 42, 3–15. 10.1002/ab.21601, PMID: 26208081

[ref26] Garofalo C. Velotti P. (2017). Negative emotionality and aggression in violent offenders: the moderating role of emotion dysregulation. J. Crime Justice 51, 9–16. 10.1016/j.jcrimjus.2017.05.015

[ref27] Garofalo C. Velotti P. Zavattini G. C. (2018). Emotion regulation and aggression: the incremental contribution of alexithymia, impulsivity, and emotion dysregulation facets. Psychol. Violence 8:470. 10.1037/vio0000141

[ref28] Garofalo C. Wright A. G. (2017). Alcohol abuse, personality disorders, and aggression: the quest for a common underlying mechanism. Aggress. Violent Behav. 34, 1–8. 10.1016/j.avb.2017.03.002

[ref29] Gillespie S. M. Garofalo C. Velotti P. (2018). Emotion regulation, mindfulness, and alexithymia: specific or general impairments in sexual, violent, and homicide offenders? J. Crime Justice 58, 56–66. 10.1016/j.jcrimjus.2018.07.006

[ref30] Gratz K. L. (2003). Risk factors for and functions of deliberate self-harm: an empirical and conceptual review. Clin. Psychol. 10, 192–205. 10.1093/clipsy.bpg022

[ref31] Gratz K. L. Gunderson J. G. (2006). Preliminary data on an acceptance-based emotion regulation group intervention for deliberate self-harm among women with borderline personality disorder. Behav. Ther. 37, 25–35. 10.1016/j.beth.2005.03.002, PMID: 16942958

[ref32] Gratz K. L. Levy R. Tull M. T. (2012). Emotion regulation as a mechanism of change in an acceptance-based emotion regulation group therapy for deliberate self-harm among women with borderline personality pathology. J. Cogn. Psychother. 26, 365–380. 10.1891/0889-8391.26.4.365

[ref33] Gratz K. L. Roemer L. (2004). Multidimensional assessment of emotion regulation and dysregulation: development, factor structure, and initial validation of the difficulties in emotion regulation scale. J. Psychopathol. Behav. Assess. 26, 41–54. 10.1023/B:JOBA.0000007455.08539.94

[ref34] Gratz K. L. Roemer L. (2008). The relationship between emotion dysregulation and deliberate self-harm among female undergraduate students at an urban commuter university. Cogn. Behav. Ther. 37, 14–25. 10.1080/16506070701819524, PMID: 18365795

[ref35] Gratz K. L. Rosenthal M. Z. Tull M. T. Lejuez C. W. Gunderson J. G. (2006). An experimental investigation of emotion dysregulation in borderline personality disorder. J. Abnorm. Psychol. 115, 850–855. 10.1037/0021-843X.115.4.850, PMID: 17100543

[ref36] Gratz K. L. Tull M. T. (2010). The relationship between emotion dysregulation and deliberate self-harm among inpatients with substance use disorders. Cogn. Ther. Res. 34, 544–553. 10.1007/s10608-009-9268-4, PMID: 21132101 PMC2996045

[ref37] Gratz K. L. Tull M. T. (2011). Extending research on the utility of an adjunctive emotion regulation group therapy for deliberate self-harm among women with borderline personality pathology. Personal. Disord. 2, 316–326. 10.1037/a0022144 22448804

[ref38] Gross J. J. (2002). Emotion regulation: Affective, cognitive, and social consequences. Psychophysiology 39, 281–291. 10.1017/S0048577201393198 12212647

[ref83] Hawton K. (2002). Studying survivors of nearly lethal suicide attempts: an important strategy in suicide research. Suicide Life Threat. Behav. 32, 76–84. 10.1521/suli.32.1.5.76.24215 11924699

[ref39] Jutengren G. Kerr M. Stattin H. (2011). Adolescents’ deliberate self-harm, interpersonal stress, and the moderating effects of self-regulation: a two-wave longitudinal analysis. J. Sch. Psychol. 49, 249–264. 10.1016/j.jsp.2010.11.001, PMID: 21530766

[ref40] Kip H. Bouman Y. H. Kelders S. M. van Gemert-Pijnen L. J. (2018). eHealth in treatment of offenders in forensic mental health: a review of the current state. Front. Psychol. 9:42. 10.3389/fpsyt.2018.00042, PMID: 29515468 PMC5826338

[ref41] Klein Tuente S. Bogaerts S. Bulten E. Keulen-de Vos M. Vos M. Bokern H.. (2020). Virtual reality aggression prevention therapy (VRAPT) versus waiting list control for forensic psychiatric inpatients: a multicenter randomized controlled trial. J. Clin. Med. 9:2258. 10.3390/jcm9072258, PMID: 32708637 PMC7409015

[ref42] Kleindienst N. Bohus M. Ludäscher P. Limberger M. F. Kuenkele K. Ebner-Priemer U. W.. (2008). Motives for nonsuicidal self-injury among women with borderline personality disorder. J. Nerv. Ment. Dis. 196, 230–236. 10.1097/NMD.0b013e3181663026, PMID: 18340259

[ref43] Klonsky E. D. (2007). The functions of deliberate self-injury: a review of the evidence. Clin. Psychol. Rev. 27, 226–239. 10.1016/j.cpr.2006.08.002, PMID: 17014942

[ref44] Klonsky E. D. (2009). The functions of self-injury in young adults who cut themselves: clarifying the evidence for affect-regulation. Psychiatry Res. 166, 260–268. 10.1016/j.psychres.2008.02.008, PMID: 19275962 PMC2723954

[ref45] Klonsky E. D. Glenn C. R. (2009). Assessing the functions of non-suicidal self-injury: psychometric properties of the inventory of statements about self-injury (ISAS). J. Psychopathol. Behav. Assess. 31, 215–219. 10.1007/s10862-008-9107-z, PMID: 29269992 PMC5736316

[ref46] Klonsky E. D. May A. M. Glenn C. R. (2013). The relationship between nonsuicidal self-injury and attempted suicide: converging evidence from four samples. J. Abnorm. Psychol. 122, 231–237. 10.1037/a0030278, PMID: 23067259

[ref47] Kun B. Demetrovics Z. (2010). Emotional intelligence and addictions: a systematic review. Subst. Use Misuse 45, 1131–1160. 10.3109/10826080903567855 20441455

[ref48] Lanza M. L. Kayne H. L. Hicks C. Milner J. (1994). Environmental characteristics related to patient assault. Issues Ment. Health Nurs. 15, 319–335. 10.3109/01612849409009393 7829320

[ref49] Laporte N. Ozolins A. Westling S. Westrin Å. Wallinius M. (2021). Clinical characteristics and self-harm in forensic psychiatric patients. Front. Psychol. 12:1277. 10.3389/fpsyt.2021.698372 PMC8365140 34408680

[ref50] Liebling A. (1993). Suicides in Prison. Crim. Justice Matters. 14:22. 10.1080/09627259308552656

[ref51] Lindholm T. Bjärehed J. Lundh L. G. (2011). Functions of nonsuicidal self-injury among young women in residential care: a pilot study with the Swedish version of the inventory of statements about self-injury. Cogn. Behav. Ther. 40, 183–189. 10.1080/16506073.2011.565791, PMID: 21877957

[ref52] Linehan M. M. (1993). Cognitive-Behavioral Treatment of Borderline Personality Disorder. New York: Guilford.

[ref53] Mann J. J. Arango V. A. Avenevoli S. Brent D. A. Champagne F. A. Clayton P.. (2009). Candidate Endophenotypes for genetic studies of suicidal behavior. Biol. Psychiatry 65, 556–563. 10.1016/j.biopsych.2008.11.021, PMID: 19201395 PMC3271953

[ref54] McCann R. A. Ball E. M. Ivanoff A. (2000). DBT with an inpatient forensic population: the CMHIP forensic model. Cogn. Behav. Pract. 7, 447–456. 10.1016/S1077-7229(00)80056-5

[ref55] McHugh C. M. Lee R. S. C. Hermens D. F. Corderoy A. Large M. Hickie I. B. (2019). Impulsivity in the self-harm and suicidal behavior of young people: a systematic review and meta-analysis. J. Psychiatr. Res. 116, 51–60. 10.1016/j.jpsychires.2019.05.012, PMID: 31195164

[ref56] Mikolajczak M. Petrides K. V. Hurry J. (2009). Adolescents choosing self-harm as an emotion regulation strategy: the protective role of trait emotional intelligence. Br. J. Clin. Psychol. 48, 181–193. 10.1348/014466508X386027, PMID: 19054434

[ref57] Moeller F. G. Barratt E. S. Dougherty D. M. Schmitz J. M. Swann A. C. (2001). Psychiatric aspects of impulsivity. Am. J. Psychiatry 158, 1783–1793. 10.1176/appi.ajp.158.11.1783, PMID: 11691682

[ref58] Muehlenkamp J. J. Xhunga N. Brausch A. M. (2018). Self-injury age of onset: a risk factor for NSSI severity and suicidal behavior. Arch. Suicide Res. 23, 551–563. 10.1080/13811118.2018.1486252 29952739 PMC8425284

[ref59] Neumann A. van Lier P. A. C. Gratz K. L. Koot H. M. (2010). Multidimensional assessment of emotion regulation difficulties in adolescents using the difficulties in emotion regulation scale. Assessment 17, 138–149. 10.1177/1073191109349579, PMID: 19915198

[ref60] Nock M. K. (2008). Actions speak louder than words: an elaborated theoretical model of the social functions of self-injury and other harmful behaviors. Appl. Prev. Psychol. 12, 159–168. 10.1016/j.appsy.2008.05.002, PMID: 19122893 PMC2574497

[ref61] O’Donnell O. House A. Waterman M. (2015). The co-occurrence of aggression and self-harm: systematic literature review. J. Affect. Disord. 175, 325–350. 10.1016/j.jad.2014.12.051, PMID: 25665494

[ref62] Podvoll E. M. (1969). Self-mutilation within a hospital setting: a study of identity and social compliance. Br. J. Med. Psychol. 42:13. 10.1111/j.2044-8341.1969.tb02073.x, PMID: 5808712

[ref63] Puzzo I. Sedgwick O. Kelly R. Greer B. Kumari V. Guðjónsson G.. (2019). Attention problems predict risk of violence and rehabilitative engagement in mentally disordered offenders. Front. Psychol. 10:279. 10.3389/fpsyt.2019.00279, PMID: 31133891 PMC6514136

[ref64] Roberton T. Daffern M. Bucks R. S. (2012). Emotion regulation and aggression. Aggress. Violent Behav. 17, 72–82. 10.1016/j.avb.2011.09.006

[ref65] Roberton T. Daffern M. Bucks R. S. (2014). Maladaptive emotion regulation and aggression in adult offenders. Psychol. Crim. Law 20, 933–954. 10.1080/1068316X.2014.893333

[ref66] Roberton T. Daffern M. Bucks R. S. (2015). Beyond anger control: difficulty attending to emotions also predicts aggression in offenders. Psychol. Violence 5, 74–83. 10.1037/a0037214

[ref67] Rothbart M. K. Ziaie H. O’Boyle C. G. (1992). Self-regulation and emotion in infancy. New Dir. Child Dev. 55, 7–23. 10.1002/cd.23219925503 1608516

[ref68] Rottenberg J. Gross J. J. (2007). Emotion and emotion regulation: a map for psychotherapy researchers. Clin. Psychol. 14, 323–328. 10.1111/j.1468-2850.2007.00093.x

[ref69] Salters-Pedneault K. Roemer L. Tull M. Rucker L. Mennin D. S. (2006). Evidence of broad deficits in emotion regulation associated with chronic worry and generalized anxiety disorder. Cogn. Ther. Res. 30, 469–480. 10.1007/s10608-006-9055-4

[ref70] Scott J. P. DiLillo D. Maldonado R. C. Watkins L. E. (2015). Negative urgency and emotion regulation strategy use: associations with displaced aggression. Aggress. Behav. 41, 502–512. 10.1002/ab.21588, PMID: 25753818

[ref71] Sherwood N. E. Crowther J. H. Wills L. Ben-Porath Y. S. (2000). The perceived function of eating for bulimic, subclinical bulimic, and non-eating disordered women. Behav. Ther. 31, 777–793. 10.1016/S0005-7894(00)80044-1

[ref72] Smith G. T. Cyders M. A. (2016). Integrating affect and impulsivity: the role of positive and negative urgency in substance use risk. Drug Alcohol Depend. 163, S3–S12. 10.1016/j.drugalcdep.2015.08.038, PMID: 27306729 PMC4911536

[ref73] Svensson I. Fälth L. Persson B. (2015). Reading level and the prevalence of a dyslexic profile among patients in a forensic psychiatric clinic. J. Forens. Psychiatry Psychol. 26, 532–550. 10.1080/14789949.2015.1037329

[ref74] Swedish National Forensic Psychiatric Register, RättspsyK (2020). Annual report 2020. Gothenburg: Swedish National Forensic Psychiatric Register.

[ref75] Tuente S. K. Bogaerts S. Van Ijzendoorn S. Veling W. (2018). Effect of virtual reality aggression prevention training for forensic psychiatric patients (VRAPT): study protocol of a multi-center RCT. BMC Psychiatry 18:251. 10.1186/s12888-018-1830-8, PMID: 30081863 PMC6091200

[ref76] Turner B. J. Chapman A. L. Layden B. K. (2012). Intrapersonal and interpersonal functions of non suicidal self-injury: associations with emotional and social functioning. Suicide Life Threat. Behav. 42, 36–55. 10.1111/j.1943-278X.2011.00069.x 22276747

[ref77] Velotti P. Garofalo C. (2015). Personality styles in a non-clinical sample: the role of emotion dysregulation and impulsivity. Personal. Individ. Differ. 79, 44–49. 10.1016/j.paid.2015.01.046

[ref78] Velotti P. Garofalo C. Petrocchi C. Cavallo F. Popolo R. Dimaggio G. (2016). Alexithymia, emotion dysregulation, impulsivity and aggression: a multiple mediation model. Psychiatry Res. 237, 296–303. 10.1016/j.psychres.2016.01.025, PMID: 26803364

[ref79] Vigil-Colet A. Ruiz-Pamies M. Anguiano-Carrasco C. Lorenzo-Seva U. (2012). The impact of social desirability on psychometric measures of aggression. Psicothema 24, 310–315. PMID: 22420362

[ref80] Yoo S. H. Matsumoto D. LeRoux J. A. (2006). The influence of emotion recognition and emotion regulation on intercultural adjustment. Int. J. Intercult. Relat. 30, 345–363. 10.1016/j.ijintrel.2005.08.006

[ref81] Zeman J. Cassano M. Perry-Parrish C. Stegall S. (2006). Emotion regulation in children and adolescents. J. Dev. Behav. Pediatr. 27, 155–168. 10.1097/00004703-200604000-00014, PMID: 16682883

[ref82] Zeman J. Shipman K. (1996). Children’s expression of negative affect: reasons and methods. Dev. Psychol. 32, 842–849. 10.1037/0012-1649.32.5.842

